# Performance comparison of stress hyperglycemia ratio for predicting fatal outcomes in patients with thrombolyzed acute ischemic stroke

**DOI:** 10.1371/journal.pone.0297809

**Published:** 2024-01-31

**Authors:** Sarawut Krongsut, Chatchon Kaewkrasaesin

**Affiliations:** 1 Division of Neurology, Department of Internal Medicine, Saraburi Hospital, Saraburi, Thailand; 2 Division of Endocrinology, Diabetes and Metabolism, Faculty of Medicine Taksin hospital, Medical Service Department, Bangkok Metropolitan Administration, Bangkok, Thailand; 3 Diabetes and metabolic care center, Taksin Hospital, Medical Service Department, Bangkok Metropolitan Administration, Bangkok, Thailand; Foshan Sanshui District People’s Hospital, CHINA

## Abstract

**Background:**

The stress hyperglycemia ratio (SHR), a newly developed metric, is used to assess adverse outcomes in patients with acute ischemic stroke (AIS). However, the relationship between SHR and fatal outcomes (in-hospital mortality [IHM], malignant cerebral edema [MCE], symptomatic intracerebral hemorrhage [sICH], 3-month mortality, and poor functional outcome) in AIS patients receiving recombinant tissue plasminogen activator (rt-PA) treatment is unclear, and determining the optimal threshold remains incomplete.

**Materials and methods:**

We retrospectively enrolled a total of 345 AIS patients treated with rt-PA during 2015–2022 and collected data on various glucose metrics, including different types of SHR, glycemic gap (GG), random plasma glucose (RPG), fasting plasma glucose (FPG), and hemoglobin A1c (HbA1c). SHR and GG were calculated using these equations: SHR1, [FPG]/[HbA1c]; SHR2, [admission RPG]/[HbA1c]; SHR3, FPG/[(1.59 × HbA1c)−2.59]; SHR4, [admission RPG]/[(1.59 × HbA1c)−2.59]; GG, admission RPG − [(1.59 × HbA1c)−2.59]. We used multivariable logistic regression analysis (MVLR) to identify the association between different glucose metrics and outcomes while comparing their predictive values.

**Results:**

SHR1 had the greatest predictive power and a more significant correlation with fatal outcomes than other continuous glucose metrics. The area under the curve of the SHR1 for IHM, MCE, and sICH, 3-month mortality, and poor functional outcome were 0.75, 0.77, 0.77, 0.76, and 0.73, respectively. SHR1 (per 1-point increases) was independently associated with IHM (Odds ratios [ORs] = 5.80; 95% CI [1.96, 17.17]; *p* = 0.001), MCE (ORs = 4.73; 95% CI [1.71, 13.04]; *p* = 0.003), sICH (ORs = 4.68, 95% CI [1.48–14.82]; *p* = 0.009), 3-month mortality (ORs = 10.87; 95% CI [3.56, 33.21]; *p*<0.001), and 3-month poor functional outcome (ORs = 8.05; 95% CI [2.77, 23.39]; p<0.001) after adjustment in MVLR. In subgroup analysis, elevated SHR1 was associated with fatal outcomes in patients with non-diabetes, SBP≥ 180 mmHg, and NIHSS <16.

**Conclusion:**

SHR1 demonstrates an independent association with fatal outcomes in AIS patients treated with rt-PA, exhibiting superior predictive ability over other glucose metrics.

## 1. Introduction

Stroke is a prevalent neurological condition and a primary global cause of death, resulting in approximately 6 million annual fatalities [1]. Stroke is the leading cause of death in Thailand, accounting for over 250,000 new cases and 50,000 annual fatalities [2]. Recombinant tissue plasminogen activator (rt-PA) is recommended as a safe and effective treatment [3]. Elevated blood sugar in 40–50% of acute stroke patients may exacerbate ischemic injury through heightened oxidative stress, endothelial dysfunction, and impaired fibrinolysis, resulting in larger infarctions, worse clinical outcomes, and increased mortality rates. [4, 5]. Stress hyperglycemia (SH) refers to transient hyperglycemia in the context of illness accompanied by diabetes mellitus (DM) or non-DM. Recently, Roberts et al. [6] introduced the stress hyperglycemia ratio (SHR) to evaluate SH. Hemoglobin A1c (HbA1c), a stable indicator, was used to assess glycemic management in DM patients over three months. SHR is calculated by dividing the admission glucose concentration by the estimated average glucose concentration derived from HbA1c [7]. Different studies employed the glucose/HbA1c ratio to define SHR, aiming for its practical use in clinical settings [8–10].

Poor outcomes and symptomatic intracerebral hemorrhage (sICH) in acute ischemic stroke (AIS) patients treated with rt-PA were associated with hyperglycemia. According to the American Diabetes Association, patients were classified as DM, newly diagnosed DM, or experiencing transient hyperglycemia during hospitalization. The definition of SH remains unclear, but an abrupt increase in plasma glucose levels above the average blood glucose level serves as a reliable indicator [6, 11]. Two types of biological markers for SH, SHR and glycemic gap (GG), have been developed to represent SH [11]. Recently, the SHR, a ratio of plasma glucose level to HbA1c, has emerged as a prognostic biomarker for poor outcomes in AIS patients receiving rt-PA treatment.

Although different SHR equations effectively predicted unfavorable outcomes or critical illness in AIS patients [12], the optimal threshold of SHR for assessing SH and predicting fatal outcomes (in-hospital mortality [IHM], malignant cerebral edema [MCE], sICH, 3-month mortality, and poor functional outcome) has not been definitively confirmed. Limited data currently exists regarding the comparative predictive value of various types of SHR, GG, absolute plasma glucose, and HbA1c in predicting fatal outcomes in AIS patients treated with rt-PA. Hence, this study aims to explore the predictive performance, optimal thresholds, and association between these variables in predicting fatal outcomes.

## 2. Methods

### 2.1. Study population

We conducted a retrospective observational cohort study by collecting data on 345 AIS patients who were treated with intravenous rt-PA at Saraburi Hospital, a stroke referral center of a provincial hospital in Thailand, between January 1, 2015 and July 31, 2022. Treatment involved administering intravenous rt-PA following the 2019 AIS management guideline [13]. Inclusion criteria: (1) age ≥ 18 years; (2) AIS within 4.5 hours of the last known normal; (3) acute anterior circulation ischemic stroke; and (4) rt-PA treatment only. Exclusion criteria: (1) minor stroke; (2) pregnancy; (3) ICH or infarction > 1/3 the middle cerebral artery (MCA) territory; (4) referred patients with unattainable follow-up; (5) missing data: National Institutes of Health Stroke Scale (NIHSS), non-contrast computed tomography (NCCT) imaging, and laboratory results. AIS patients receiving EVT were not included in this study. AIS patients suspected of large vessel occlusion (LVO) were not transferred for EVT during the study period due to limitations in Thailand’s public health coverage, causing difficulties in accessing this treatment. All patients stayed in the hospital, regardless of LVO. Data comprising clinical and imaging information were retrieved from our electronic medical records, with diagnoses established using the International Classification of Diseases, 10th Revision codes (I63). The data were fully anonymized before we accessed them, and the ethics committee waived the requirement for informed consent. We don’t collect patient-identifying information, including hospital numbers, admission numbers, identity card numbers, or birthdates. The study received ethical approval from the human research ethics committee of Saraburi Hospital on January 30, 2023 (Certificate No. EC004/2566). We accessed the data for research purposes on February 5, 2023.

### 2.2 Data collection

Demographic data, encompassing age, gender, initial clinical presentation, medical history, laboratory investigations, time from symptom onset to treatment, admission blood pressure, Trial of Org 10172 in Acute Stroke Treatment (TOAST) classification, and neuroimaging results, were collected. The extent of early ischemic changes was evaluated using the Alberta Stroke Program Early Computed Tomography Score (ASPECTS). Certified neurologists utilized NIHSS to evaluate stroke severity upon admission. The plasma glucose level on admission was measured before thrombolytic treatment. The history of DM was established by reviewing the patient’s medical diagnosis and their antidiabetic drug usage record.

### 2.3 Collection and processing of blood samples and laboratory tests

#### 2.3.1 Patient preparation

We collected fasting plasma glucose (FPG) and HbA1c after an 8–14 hour fasting but not exceeding 16 hours, to avoid starvation, with a morning collection between 6:00 a.m. and 10:00 a.m. Random plasma glucose (RPG) was measured upon hospital arrival regardless of the time since the last meal.

#### 2.3.2 Methods for collecting and submitting specimens

Blood collection tubes with anticoagulants like sodium fluoride/potassium oxalate were used to collect 2 cc of blood for plasma glucose testing, and tubes with ethylene diamine tetra acetate were used for HbA1c testing. The samples were analyzed within 45 minutes of collection, with results reported within 1 hour. Blood samples for plasma glucose were analyzed using an automated analyzer (Beckman Coulter DxC 700 AU) with the Beckman Coulter glucose reagent. Blood samples for HbA1c were analyzed using an automated analyzer (Mindray BS-820M) with the HbA1c reagent.

We collected data on various glucose metrics, including different types of SHR, GG, absolute blood glucose (RPG and FPG), and HbA1c. SHR and GG were calculated using the following equations: SHR1 [14], [FPG (mmol/L)]/[HbA1c (%)]; SHR2 [15], [admission RPG (mmol/L)]/[HbA1c (%)]; SHR3 [12], FPG (mmol/L)/[(1.59 × HbA1c)−2.59]; SHR4 [16], [admission RPG (mmol/L)]/[(1.59 × HbA1c)−2.59]; and GG [17], admission RPG − [(1.59 × HbA1c)−2.59]. In this study, neither the treating physicians nor the nurses were involved in measuring the SHR and GG values. We have provided laboratory protocols hosted on the protocols.io platform at the following link: https://www.protocols.io/view/performance-comparison-of-stress-hyperglycemia-rat-bp2l6xr8klqe/v1.

### 2.4 Outcomes assessment

The primary outcome of the study was IHM defined as patients with thrombolyzed AIS who died in the hospital. The secondary outcomes were MCE, sICH, 3-month mortality, and poor functional outcome. The diagnostic criteria for malignant cerebral edema (MCE) were as follows: (i) acute complete MCA infarction with parenchymal hypodensity covering at least 50% of the MCA territory, along with sulcal effacement and lateral ventricle compression; (ii) excessive midline shift exceeding 5 mm and obliteration of basal cisterns; and (iii) neurological deterioration was characterized by an increase in NIHSS score (more than 2 points) and a decline in consciousness level (at least 1 point in item 1A of the NIHSS assessment) [18]. Based on the National Institute of Neurological Disorders and Stroke criteria, sICH was defined as any deterioration in NIHSS score or mortality within 7 days of thrombolysis initiation, along with the presence of any type of intracerebral hemorrhage on posttreatment imaging. [19]. The survival status was determined by utilizing mortality data derived from electronic medical records and death certificates, which were supplied by local municipal authorities for each study participant. 3-month mortality referred to death within 90 days regardless of causes, and 3-month poor functional outcome was defined as mRS > 2 at 90 days after a stroke All included patients were followed up through telephone interviews conducted by stroke-trained nurses and/or physical therapy staff 90 days after the stroke. NCCT scans were done within 4.5 hours of symptom onset and repeated at 24 hours post-thrombolysis. An emergency NCCT would be performed for deteriorating neurological deficits.

### 2.5 Statistical analysis

We analyzed the data using Stata version 17 (StataCorp, Lakeway, Texas 77845, USA) and considered a two-tailed *p*-value <0.05 statistically significant. Continuous variables with a normal distribution were summarized using mean and standard deviation, while those non-normal distributed variables were described using median and interquartile range (IQR). Categorical variables were presented as frequencies and percentages. Statistical tests such as t-test, Mann-Whitney U-test, and chi-squared test were used to compare differences between the two groups, depending on variables. The predictive potential of different admission glucose measures (SHR1, SHR2, SHR3, SHR4, GG, FPG, admission RPG, and HbA1c) for fatal outcomes (IHM, MCE, sICH, 3-month mortality, and poor functional outcome) was evaluated. Sensitivity, specificity, positive and negative predictive values, likelihood ratios of positive and negative, and accuracy were analyzed. The multivariable logistic regression (MVLR) model, considering relevant factors, was employed to determine odds ratios (ORs) and 95% confidence intervals (CIs) for predicting fatal outcomes. We selected variables for adjustment in the MVLR model based on previous background knowledge. For IHM, the crude model represents univariable analysis. In model A, we adjusted for age and sex. In model B, we adjusted for variables in model A plus TOAST classification, NIHSS, and baseline ASPECTS ≤6. In model C, we additionally adjusted for comorbidities (DM, chronic kidney disease [CKD], myocardial infarction [MI], congestive heart failure [CHF], mRS, history of cancer), systolic blood pressure (SBP), and diastolic blood pressure (DBP) to assess the relationship between glucose metrics and fatal outcomes.

To maintain the integrity of variable relationships, we assessed the impact of the approach in the MVLR model. We examined confounding factors and their effect on SHR and conducted a subgroup analysis to explore the multiplicative interaction. The receiver operating characteristic (ROC) curves for glucose metrics were compared using a nonparametric method [20], and the optimal cut-off value of glucose metrics at admission was determined using the Youden’s index method for predicting fatal outcomes.

## 3. Results

**Fig 1:**
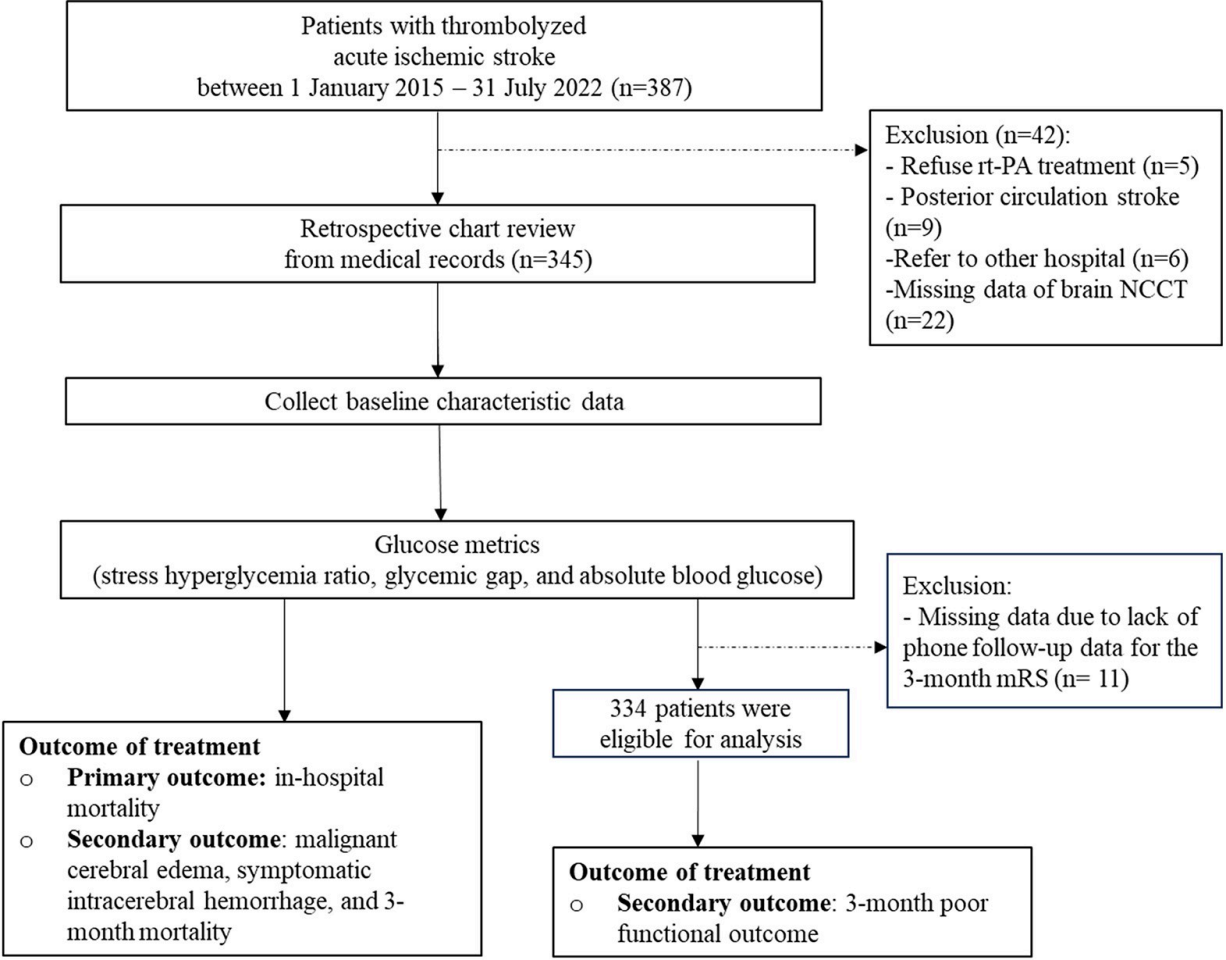
The patient flow chart.

In this retrospective study, a total of 387 patients were diagnosed with thrombolysis-indicated AIS during January 1, 2015 to July 31, 2022. Five patients refused rt-PA treatment, nine patients were diagnosed with posterior circulation ischemic stroke, six patients were referred to another hospital, and 22 patients with missing brain CT data were excluded. The remaining 345 patients were included for analysis in this cohort. However, 11 patients (3.19%) had missing data on the 3-month mRS evaluation for assessing poor functional outcomes. (Fig 1)

### 3.1 Baseline characteristics

According to Table 1, the mean age of the patients was 61.8 ± 15.2 years, and 53.04% of the patients were male, and the median NIHSS score was 11 points (IQR: 8–17). Among the 345 patients included in the study, 65 (18.84%) died during hospitalization, 52 (15.07%) developed MCE, 42 (12.17%) developed sICH, 83 (24.06%) had 3-month mortality, and 138 (41.32%) had 3-month poor functional outcome. The median hospitalization duration was 5 days (IQR: 3–9). Median time from admission to sICH was 2 days (IQR: 1–3), and to MCE was 5 days (IQR: 2–6). Patients who did not survive were more likely to be aged ≥70 years, have a history of atrial fibrillation, CHF, CKD, history of malignancy, swallowing dysfunction, aphasia, neglect, cranial nerve disorder, gaze paresis, preexisting disability (mRS ≥2), higher SBP and DBP, NIHSS ≥16, large artery atherosclerosis or cardioembolic stroke, and receive intravenous antihypertensive treatment before rt-PA, compared to those who survived. Remarkably, patients who developed IHM had significantly higher SHR1, SHR2, SHR3, SHR4, GG, FPG, and admission RPG than those who survived.

**Table 1:** Comparison of the baseline characteristics between patients with IHM and survived.

Characteristic	All patients (n = 345)	Primary outcomes		*p*-value
		IHM (n = 64)	survived (n = 281)	
Age, years				
18–59	151 (43.8%)	20 (31.3%)	131 (46.6%)	0.001
60–69	81 (23.5%)	11 (17.2%)	70 (24.9%)	
70–79	62 (18%)	14 (21.9%)	48 (17.1%)	
> = 80	51 (14.8%)	19 (29.7%)	32 (11.4%)	
Gender				
Male	183 (53%)	33 (51.6%)	150 (53.4%)	0.793
Female	162 (47%)	31 (48.4%)	131 (46.6%)	
Vascular risk factors and comorbidities				
Smoking	123 (35.7%)	19 (29.7%)	104 (37%)	0.27
Alcohol	142 (41.2%)	27 (42.2%)	115 (40.9%)	0.853
Prior use antiplatelet	66 (19.1%)	16 (25%)	50 (17.8%)	0.186
Prior stroke	41 (11.9%)	9 (14.1%)	32 (11.4%)	0.551
Atrial fibrillation	102 (29.6%)	36 (56.3%)	66 (23.5%)	<0.001
MI	29 (8.4%)	9 (14.1%)	20 (7.1%)	0.071
CHF	37 (10.7%)	12 (18.8%)	25 (8.9%)	0.022
Valvular heart disease	22 (6.4%)	4 (6.3%)	18 (6.4%)	0.963
Diabetes mellitus	93 (27%)	16 (25%)	77 (27.4%)	0.696
Hypertension	243 (70.4%)	48 (75%)	195 (69.4%)	0.375
Chronic kidney disease	44 (12.8%)	13 (20.3%)	31 (11%)	0.045
Dyslipidemia	141 (40.9%)	25 (39.1%)	116 (41.3%)	0.745
Gout	11 (3.2%)	4 (6.3%)	7 (2.5%)	0.122
History of malignancy	8 (2.3%)	4 (6.3%)	4 (1.4%)	0.021
History of renal replacement therapy	5 (1.4%)	2 (3.1%)	3 (1.1%)	0.214
Clinical presentation				
Hemiparesis	341 (98.8%)	63 (98.4%)	278 (98.9%)	0.739
Dysarthria	275 (79.7%)	53 (82.8%)	222 (79%)	0.494
Swallowing dysfunction	129 (37.4%)	51 (79.7%)	78 (27.8%)	<0.001
Ataxia	37 (10.7%)	7 (10.9%)	30 (10.7%)	0.951
Hemianopia	23 (6.7%)	6 (9.4%)	17 (6%)	0.336
Aphasia	132 (38.3%)	46 (71.9%)	86 (30.6%)	<0.001
Neglect	62 (18%)	19 (29.7%)	43 (15.3%)	0.007
Cranial nerve disorder	12 (3.5%)	5 (7.8%)	7 (2.5%)	0.036
Gaze paresis	112 (32.5%)	49 (76.6%)	63 (22.4%)	<0.001
Pre-stroke functional status, n (%)				
Preexisting disability (mRS)				
0	320 (92.8%)	49 (76.6%)	271 (96.4%)	<0.001
1	6 (1.7%)	1 (1.6%)	5 (1.8%)	
2–3	19 (5.5%)	14 (21.9%)	5 (1.8%)	
Time to rt-PA, hour				
< 3hour	233 (67.5%)	39 (60.9%)	194 (69%)	0.212
3–4.5 hour	112 (32.5%)	25 (39.1%)	87 (31%)	
Blood pressure at admission, mmHg				
SBP, mmHg	159.2 ± 28.88	168.61 ± 30.17	157.06 ± 28.2	0.004
DBP, mmHg	92.34 ± 19.16	98.56 ± 17.63	90.93 ± 19.24	0.004
NIHSS at admission				
5–15	232 (67.2%)	10 (15.6%)	222 (79%)	<0.001
16–20	76 (22%)	33 (51.6%)	43 (15.3%)	
>20	37 (10.7%)	21 (32.8%)	16 (5.7%)	
TOAST classification				
Large-artery atherosclerosis	76 (22%)	17 (26.6%)	59 (21%)	<0.001
Cardioembolism	119 (34.5%)	45 (70.3%)	74 (26.3%)	
Small-vessel Occlusion	134 (38.8%)	1 (1.6%)	133 (47.3%)	
Stroke of other determined etiology	9 (2.6%)	1 (1.6%)	8 (2.8%)	
Stroke of undetermined etiology	7 (2%)	0 (0%)	7 (2.5%)	
Hospital stay, days	5 (3, 9)	8 (3, 22)	5 (4, 8)	0.008
Antihypertensive before rt-PA	97 (28.1%)	36 (56.3%)	61 (21.7%)	<0.001
Laboratory				
WBC (x 10^3^cells/mm^3^)–median (IQR)	8500 (7100, 10400)	8750 (7300, 11200)	8500 (7000, 10300)	0.312
NLR–median (IQR)	2.21 (1.53, 3.67)	2.78 (1.76, 5.23)	2.17 (1.5, 3.5)	0.020
Hb (g/dL)–mean (SD)	12.55 ± 2.13	11.91 ± 2.31	12.7 ± 2.07	0.013
Hct (%)	38.19 ± 6.29	36.35 ± 7.12	38.6 ± 6.02	0.021
Platelet (x 10^3^cells/mm3)–mean (SD)	251.51 ± 81.36	237.11 ± 81.34	254.79 ± 81.15	0.117
INR–mean (SD)	0.97 ± 0.14	0.98 ± 0.17	0.97 ± 0.13	0.356
Creatinine (mg/dL)–median (IQR)	0.95 (0.78, 1.15)	1 (0.81, 1.24)	0.94 (0.78, 1.14)	0.198
Glucose metrics at admission				
SHR 1 –mean (SD)	1.11 ± 0.32	1.34 ± 0.36	1.06 ± 0.29	<0.001
SHR 2 –mean (SD)	1.27 ± 0.4	1.49 ± 0.52	1.22 ± 0.35	<0.001
SHR3 –mean (SD)	0.97 ± 0.34	1.17 ± 0.33	0.93 ± 0.32	<0.001
SHR4 –mean (SD)	1.11 ± 0.4	1.29 ± 0.43	1.07 ± 0.39	<0.001
Glycemic gap–mean (SD)	0.7 ± 2.62	2.19 ± 3.62	0.36 ± 2.21	<0.001
Fasting plasma glucose (mmol/L)–mean (SD)	6.84 ± 2.49	8.27 ± 3.08	6.51 ± 2.21	<0.001
Admission random plasma glucose (mmol/L)–mean (SD)	7.97 ± 3.83	9.35 ± 4.81	7.65 ± 3.5	<0.001
HbA1c (%)–mean (SD)	6.20 ± 1.56	6.14 ± 1.34	6.22 ± 1.61	0.710
Workflow time				
Onset to door, min–median (IQR)	90 (60, 120)	90 (60, 130)	90 (60, 120)	0.181
Onset to treatment time, min–median (IQR)	140 (96, 182)	151.5 (97, 189.5)	135 (96, 180)	0.370
ASPECTS				
Baseline ASPECTS–median (IQR)	10 (8, 10)	7 (6, 8)	10 (9, 10)	<0.001

**Abbreviations:** ASPECTS, Alberta Stroke Program Early CT Score; CHF, congestive heart failure; CI: confidence interval; DBP, diastolic blood pressure; Glycated hemoglobin, HbA1c; Hb, hemoglobin; Hct, hematocrit; IHM, in-hospital mortality; IQR, interquartile range; MI, myocardial infarction; mRS, modified Rankin Scale; NIHSS, National Institutes of Health Stroke Scale; NLR, neutrophil lymphocyte count ratio; rt-PA, recombinant tissue plasminogen activator; SBP, systolic blood pressure; SHR, stress hyperglycemia ratio; SHR1, [FPG (mmol/L)]/[HbA1c (%)]; SHR2, [admission RPG (mmol/L)]/[HbA1c (%)]; SHR3, FPG (mmol/L)/[(1.59 × HbA1c)−2.59]; SHR4, [admission RPG (mmol/L)]/[(1.59 × HbA1c)−2.59]; SD, standard deviation; TOAST classification, trial of ORG 10172 in acute stroke treatment classification; WBC, white blood cell count.

### 3.2 Predictive value of glucose metrics and ROC curve for predicting fatal outcomes

**Fig 2:**
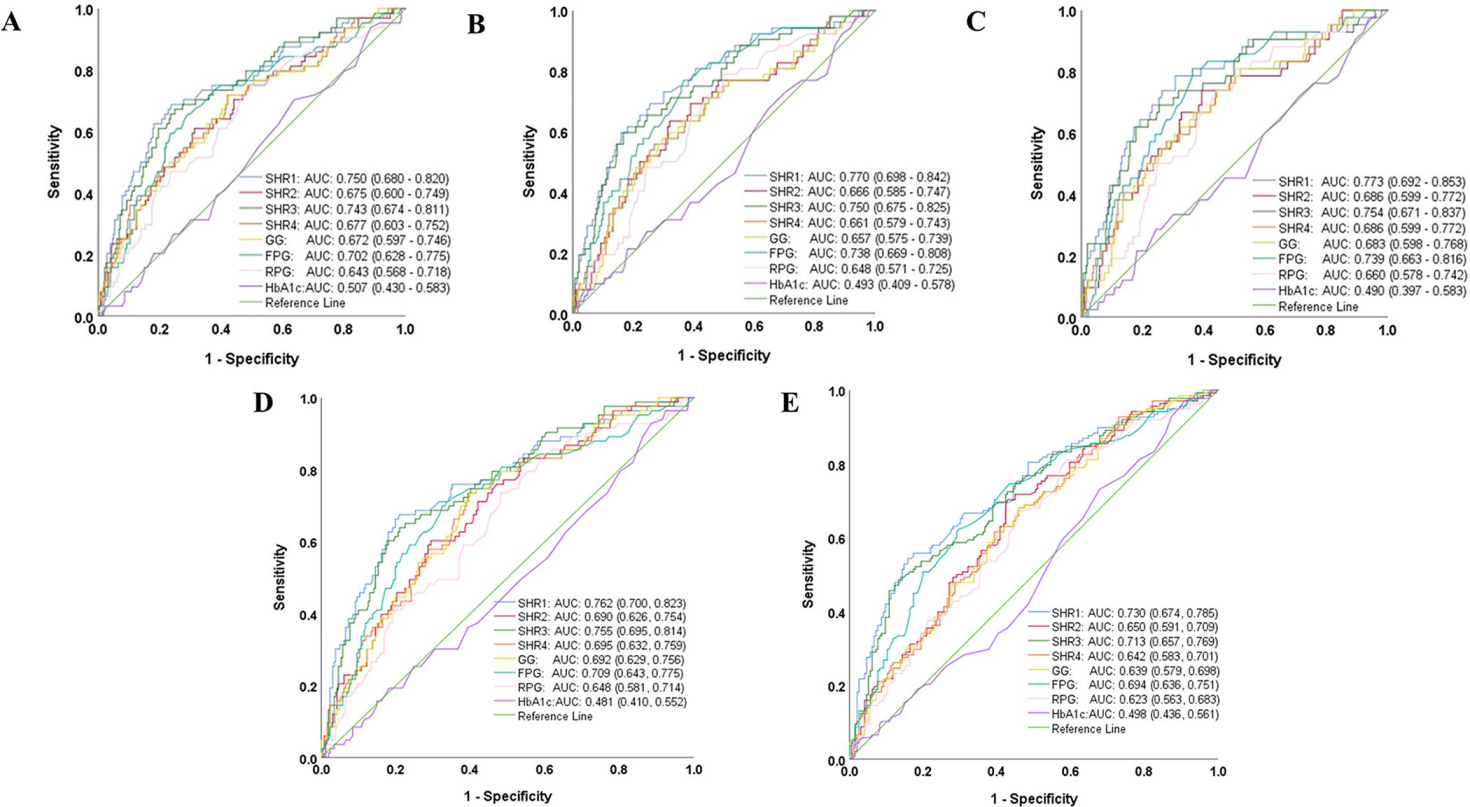
The ROC analyses of SHR, GG, absolute plasma glucose [FPG, and admission RPG], and HbA1c for predicting fatal outcomes. Dataset: (A) IHM, (B) MCE, (C) sICH, (D) 3-month mortality, and (E) 3-month poor functional outcome.

The ROC curve analysis was employed to assess the predictive efficacy of SHR1, SHR2, SHR3, SHR4, GG, FPG, admission RPG, and HbA1c in predicting fatal outcomes post-thrombolysis. SHR1 presented the highest discrimination among all 8 glucose metrics. The area under the ROC curve (AuROC) of SHR1 was 0.75 (95% CI, 0.68–0.82), 0.77 (95% CI, 0.70–0.84), 0.77 (95% CI, 0.69–0.85), 0.76 (95% CI, 0.70–0.82), and 0.73 (95% CI, 0.67–0.79) for predicting IHM, MCE, sICH, 3-month mortality, and poor functional outcome respectively (Table 2 and Fig 2A–2E). The optimal cut-off value of SHR1 for predicting IHM, MCE, and sICH was ≥1.18, ≥1.18, and ≥1.12, respectively. The sensitivity and specificity obtained were 67.2% and 76.5% for IHM and MCE, 78.6% and 69.0% for sICH, 66.3% and 79.4% for 3-month mortality, and 54.3% and 83.2% for 3-month poor functional outcome, respectively. The predictive values of glucose metrics for fatal outcomes are summarized in Table 2.

**Table 2:** AuROC analysis, optimal cutoff score, predictive values of glucose metrics at admission for predicting IHM, MCE, sICH, 3-month mortality, and 3-month poor functional outcome.

Variable	AuROC (95%CI)	Optimal cut-off	Sensitivity (95%CI)	Specificity (95%CI)	PPV (95%CI)	NPV (95%CI)	LR+ (95%CI)	LR- (95%CI)	Accuracy (95%CI)
**In-hospital mortality (n = 65)**									
SHR 1	0.75 (0.68–0.82)	≥1.18	67.2 (54.3–78.4)	76.5 (71.1–81.3)	39.4 (30.2–49.3)	91.1 (86.7–94.4)	2.86 (2.18–3.75)	0.43 (0.30–0.61)	74.8 (69.9–79.3)
SHR 2	0.68 (0.60–0.75)	≥1.26	59.4 (46.4–71.5)	68.7 (62.9–74.1)	30.2 (22.3–39.0)	88.1 (83.1–92.1)	1.90 (1.45–2.48)	0.59 (0.44–0.80)	67.0 (61.7–71.9)
SHR3	0.74 (0.67–0.81)	≥1.01	67.2 (54.3–78.4)	75.1 (69.6–80.0)	38.1 (29.1–47.7)	90.9 (86.5–94.3)	2.70 (2.07–3.52)	0.44 (0.31–0.62)	73.6 (68.6–78.2)
SHR4	0.68 (0.60–0.75)	≥1.03	68.8 (55.9–79.8)	58.0 (52.0–63.8)	27.2 (20.5–34.7)	89.1 (83.6–93.2)	1.64 (1.32–2.03)	0.54 (0.37–0.79)	60.0 (54.6–65.2)
Glycemic gap (mmol/L)	0.67 (0.60–0.75)	≥0.17	71.9 (59.2–82.4)	57.7 (51.6–63.5)	27.9 (21.2–35.4)	90.0 (84.7–94.0)	1.70 (1.38–2.08)	0.49 (0.33–0.73)	60.3 (54.9–65.5)
Fasting plasma glucose (mmol/L)	0.70 (0.63–0.78)	≥6.91	65.6 (52.7–77.1)	70.8 (65.1–76.1)	33.9 (25.6–42.9)	90.0 (85.3–93.7)	2.25 (1.74–2.9)	0.49 (0.34–0.69)	69.9 (64.7–74.7)
Admission random plasma glucose (mmol/L)	0.64 (0.57–0.72)	≥6.47	75.0 (62.6–85.0)	50.5 (44.5–56.5)	25.7 (19.6–32.6)	89.9 (84.1–94.1)	1.52 (1.26–1.82)	0.50 (0.32–0.77)	55.1 (49.7–60.4)
HbA1c (%)	0.51 (0.43–0.58)	≥5.55	70.3 (57.6–81.1)	36.3 (30.7–42.2)	20.1 (15.0–25.9)	84.3 (76.6–90.3)	1.10 (0.92–1.32)	0.82 (0.54–1.23)	42.6 (37.3–48.0)
**Malignant cerebral edema (n = 52)**									
SHR 1	0.77 (0.70–0.84)	≥1.18	67.2 (54.3–78.4)	76.5 (71.1–81.3)	39.4 (30.2–49.3)	91.1 (86.7–94.4)	2.86 (2.18–3.75)	0.43 (0.30–0.61)	73.6 (68.6–78.2)
SHR 2	0.67 (0.59–0.75)	≥1.26	59.4 (46.4–71.5)	68.7 (62.9–74.1)	30.2 (22.3–39.0)	88.1 (83.1–92.1)	1.90 (1.45–2.48)	0.59 (0.44–0.80)	67.0 (61.7–71.9)
SHR3	0.75 (0.68–0.83)	≥1.10	51.6 (38.7–64.2)	83.3 (78.4–87.4)	41.3 (30.4–52.8)	88.3 (83.8–91.9)	3.08 (2.17–4.39)	0.58 (0.45–0.75)	79.7 (75.1–83.8)
SHR4	0.66 (0.58–0.74)	≥1.15	53.1 (40.2–65.7)	73.0 (67.4–78.1)	30.9 (22.4–40.4)	87.2 (82.3–91.2)	1.96 (1.46–2.65)	0.64 (0.49–0.84)	69.9 (64.7–74.7)
Glycemic gap (mmol/L)	0.66 (0.58–0.74)	≥0.84	54.7 (41.7–67.2)	70.8 (65.1–76.1)	29.9 (21.8–39.1)	87.3 (82.2–91.3)	1.87 (1.41–2.50)	0.64 (0.48–0.85)	68.4 (63.2–73.3)
Fasting plasma glucose (mmol/L)	0.74 (0.67–0.81)	≥6.52	71.9 (59.2–82.4)	64.1 (58.1–69.7)	31.3 (23.9–39.5)	90.9 (86.0–94.5)	2.00 (1.61–2.49)	0.44 (0.29–0.66)	65.5 (60.2–70.5)
Admission random plasma glucose (mmol/L)	0.65 (0.57–0.73)	≥6.47	75.0 (62.6–85.0)	50.5 (44.5–56.5)	25.7 (19.6–32.6)	89.9 (84.1–94.1)	1.52 (1.26–1.82)	0.50 (0.32–0.77)	54.5 (49.1–59.8)
HbA1c (%)	0.49 (0.41–0.58)	≥4.65	95.3 (86.9–99.0)	4.98 (2.75–8.22)	18.6 (14.5–23.2)	82.4 (56.6–96.2)	1.00 (0.94–1.07)	0.94 (0.28–3.18)	19.4 (15.4–24.0)
**Symptomatic intracerebral hemorrhage (n = 42)**									
SHR1	0.77 (0.69–0.85)	≥1.12	78.6 (63.2–89.7)	69.0 63.4–74.1)	26.0 (18.6–34.5)	95.9 (92.3–98.1)	2.53 (2.01–3.19)	0.31 (0.17–0.56)	70.1 (65.0–74.9)
SHR 2	0.69 (0.60–0.77)	≥1.20	73.8 (58.0–86.1)	60.4 (54.6–65.9)	20.5 (14.4–27.9)	94.3 (90.1–97.1)	1.86 (1.48–2.34)	0.43 (0.26–0.73)	62.0 (56.7–67.2)
SHR3	0.75 (0.67–0.84)	≥1.10	61.9 (45.6–76.4)	82.2 (77.4–86.3)	32.5 (22.4–43.9)	94.0 (90.4–96.5)	3.47 (2.48–4.87)	0.46 (0.31–0.68)	79.7 (75.1–83.8)
SHR4	0.69 (0.60–0.77)	≥1.03	71.4 (55.4–84.3)	56.4 (50.6–62.1)	18.5 (12.9–25.4)	93.4 (88.8–96.6)	1.64 (1.30–2.06)	0.51 (0.31–0.83)	58.3 (52.9–63.5)
Glycemic gap (mmol/L)	0.68 (0.60–0.77)	≥0.45	66.7 (50.5–80.4)	63.4 (57.7–68.8)	20.1 (13.8–27.8)	93.2 (88.9–96.2)	1.82 (1.40–2.36)	0.53 (0.34–0.81)	63.8 (58.4–68.8)
Fasting plasma glucose (mmol/L)	0.74 (0.66–0.82)	≥6.63	81.0 (65.9–91.4)	63.7 (58.0–69.1)	23.6 (16.9–31.4)	96.0 (92.3–98.3)	2.23 (1.81–2.75)	0.3 (0.16–0.56)	65.8 (60.5–70.8)
Admission random plasma glucose (mmol/L)	0.66 (0.58–0.74)	≥6.47	81.0 (65.9–91.4)	49.5 (43.7–55.3)	18.2 (12.9–24.5)	94.9 (90.3–97.8)	1.60 (1.33–1.93)	0.39 (0.20–0.73)	53.3 (47.9–58.7)
HbA1c (%)	0.49 (0.40–0.58)	≥6.45	28.6 (15.7–44.6)	75.6 (70.3–80.3)	14.0 (7.4–23.1)	88.4 (83.9–92.0)	1.17 (0.70–1.96)	0.95 (0.77–1.16)	69.9 (64.7–74.7)
**3-month mortality (n = 83)**									
SHR1	0.76 (0.70–0.82)	≥1.18	66.3 (55.1–76.3)	79.4 (74.0–84.1)	50.5 (40.7–60.2)	88.1 (83.3–92.0)	3.22 (2.42–4.27)	0.43 (0.31–0.58)	76.2 (71.4–80.6)
SHR 2	0.69 (0.63–0.75)	≥1.26	59.0 (47.7–69.7)	70.6 (64.7–76.1)	38.9 (30.3–48)	84.5 (79.0–89.0)	2.01 (1.55–2.60)	0.58 (0.44–0.76)	67.8 (62.6–72.7)
SHR3	0.76 (0.70–0.81)	≥1.03	63.9 (52.6–74.1)	79.0 (73.6–83.8)	49.1 (39.3–58.9)	87.3 (82.4–91.3)	3.04 (2.29–4.05)	0.46 (0.34–0.61)	75.4 (70.5–79.8)
SHR4	0.70 (0.63–0.76)	≥1.03	71.1 (60.1–80.5)	60.7 (54.5–66.6)	36.4 (29.0–44.3)	86.9 (81.1–91. 4)	1.81 (1.48–2.22)	0.48 (0.34–0.68)	63.2 (57.9–68.3)
Glycemic gap (mmol/L)	0.69 (0.63–0.76)	≥0.17	73.5 (62.7–82.6)	60.3 (54.1–66.3)	37 (29.6–44.8)	87.8 (82.1–92.2)	1.85 (1.52–2.26)	0.44 (0.30–0.64)	63.5 (58.2–68.6)
Fasting plasma glucose (mmol/L)	0.71 (0.64–0.78)	≥6.52	71.1 (60.1–80.5)	66.4 (60.3–72.1)	40.1 (32.1–48.5)	87.9 (82.5–92.1)	2.12 (1.70–2.63)	0.44 (0.31–0.62)	67.5 (62.3–72.5)
Admission random plasma glucose (mmol/L)	0.65 (0.58–0.71)	≥6.46	73.5 (62.7–82.6)	51.9 (45.7–58.1)	32.6 (26.0–39.8)	86.1 (79.7–91.1)	1.53 (1.28–1.83)	0.51 (0.35–0.74)	57.1 (51.7–62.4)
HbA1c (%)	0.48 (0.41–0.55)	≥4.95	92.8 (84.9–97.3)	11.5 (7.9–15.9)	24.9 (20.2–30.1)	83.3 (67.2–93.6)	1.05 (0.97–1.13)	0.63 (0.27–1.46)	31 (26.2–36.2)
**3-month poor functional outcome (n = 138)**									
SHR1	0.73 (0.67–0.79)	≥1.18	54.3 (45.7–62.8)	83.2 (77.2–88.1)	69.4 (59.8–77.9)	72.1 (65.8–77.9)	3.23 (2.28–4.57)	0.55 (0.45–0 .67)	69.0 (63.8–73.8)
SHR 2	0.65 (0.59–0.71)	≥1.15	68.8 (60.4–76.4)	57.7 (50.4–64.7)	53.4 (45.8–60.9)	72.4 (64.7–79.3)	1.63 (1.33–1.98)	0.54 (0.41–0.71)	60.0 (54.6–65.2)
SHR3	0.71 (0.66–0.77)	≥1.01	53.6 (44.9–62.1)	80.6 (74.4–85.9)	66.1 (56.5–74.7)	71.2 (64.7–77.0)	2.77 (2.00–3.83)	0.58 (0 .48–0.70)	67.2 (62–72.2)
SHR4	0.64 (0.58–0.70)	≥1.03	60.1 (51.5–68.4)	60.7 (53.5–67.6)	51.9 (43.8–59.8)	68.4 (60.9–75.2)	1.53 (1.23–1.91)	0.66 (0.52–0.83)	58.6 (53.2–63.8)
Glycemic gap (mmol/L)	0.64 (0.58–0.70)	≥0.17	61.6 (52.9–69.7)	60.2 (53.0–67.1)	52.1 (44.2–60.0)	69.0 (61.5–75.8)	1.55 (1.25–1.92)	0.64 (0.50–0.81)	58.8 (53.4–64.1)
Fasting plasma glucose (mmol/L)	0.69 (0.64–0.75)	≥6.50	62.3 (53.7–70.4)	70.4 (63.5–76.7)	59.7 (51.2–67.8)	72.6 (65.7–78.8)	2.11 (1.64–2.71)	0.54 (0.42–0.68)	64.9 (59.6–70)
Admission random plasma glucose (mmol/L)	0.62 (0.56–0.68)	≥6.46	67.4 (58.9–75.1)	54.6 (47.3–61.7)	51.1 (43.6–58.6)	70.4 (62.5–77.5)	1.48 (1.22–1.80)	0.60 (0.46–0.78)	58.0 (52.6–63.2)
HbA1c (%)	0.50 (0.44–0.56)	≥5.65	20.3 (13.9–28.0)	79.1 (72.7–84.6)	40.6 (28.9–53.1)	58.5 (52.3–64.5)	0.97 (0.63–1.49)	1.01 (0.90–1.13)	47.8 (42.4–53.2)

**Abbreviations:** AuROC, area under receiver-operating-characteristic curve; CI: confidence interval; FPG, fasting plasma glucose; Glycated hemoglobin, HbA1c; GG, glycemic gap IHM, in-hospital mortality; LR+, positive likelihood ratio; LR-, negative likelihood ratio; MCE, malignant cerebral edema; NPV: negative predictive value; PPV: positive predictive value; RPG, random plasma glucose; sICH, symptomatic intracerebral hemorrhage; SHR, stress hyperglycemia ratio; SHR1, [FPG (mmol/L)]/[HbA1c (%)]; SHR2, [admission RPG (mmol/L)]/[HbA1c (%)]; SHR3, FPG (mmol/L)/[(1.59 × HbA1c)−2.59]; SHR4, [admission RPG (mmol/L)]/[(1.59 × HbA1c)−2.59]; GG, admission RPG − [(1.59 × HbA1c)−2.59].

### 3.3 Relationship between glucose metrics and fatal outcomes

#### 3.3.1 IHM

We evaluated the association between various types of glucose metrics and IHM, and found that all types of glucose metrics were determinants of IHM in all the evaluated models, except for HbA1c. SHR1 with each one-point increase was associated with IHM after adjustment for age, sex, TOAST classification, NIHSS, baseline ASPECTS ≤7, and comorbidities (DM, CKD, MI, CHF, preexisting disability, and history of malignancy) (OR = 5.80; 95% CI: 1.96, 17.17; *p* =  0.001), whereas HbA1c was not associated with the outcome by any model. Based on all evaluated models, SHR1 ≥ 1.18, SHR2 ≥1.26, SHR3 ≥ 1.01, SHR4 ≥ 1.03, GG ≥ 0.17 mmol/L, FPG ≥ 6.91 mmol/L, and admission RPG ≥ 6.47 mmol/L showed significant association with IHM after adjusting for the confounders in all models. Only HbA1c ≥ 5.55 was not associated with IHM in any of the models. Among the continuous glucose metrics, SHR1 exhibited the strongest correlation with IHM when compared to others (Table 3).

**Table 3:** Univariable and multivariable logistic regression analysis of the relationship between glucose metrics and fatal outcomes.

Predictors	Univariate		Multivariate analysis					
			Model A		Model B		Model C	
	OR (95%CI)	*p*-value	AOR (95%CI)	*p*-value	AOR (95%CI)	*p*-value	AOR (95%CI)	*p*-value
**In-hospital mortality**^†^ **(n = 65)**								
**Continuous glucose metrics at admission**								
SHR1 (per 1 point increase)	10.99 (4.68, 25.8)	<0.001	11.95 (5.01, 28.51)	<0.001	4.90 (1.69, 14.20)	0.003	5.80 (1.96, 17.17)	0.001
SHR2 (per 1 point increase)	4.20 (2.20, 8.01)	<0.001	4.24 (2.19, 8.22)	<0.001	3.24 (1.38, 7.61)	0.007	3.92 (1.57, 9.8)	0.003
SHR3 (per 1 point increase)	7.47 (3.07, 18.14)	<0.001	8.13 (3.27, 20.2)	<0.001	3.11 (1.27, 7.63)	0.013	3.17 (1.30, 7.75)	0.011
SHR4 (per 1 point increase)	3.57 (1.70, 7.49)	0.001	3.51 (1.65, 7.45)	0.001	2.42 (1.18, 4.99)	0.016	2.38 (1.13, 5.02)	0.023
GG (per 1 mmol/L increase)	1.27 (1.14, 1.40)	<0.001	1.27 (1.14, 1.42)	<0.001	1.20 (1.05, 1.38)	0.009	1.27 (1.08, 1.49)	0.004
FPG (per 1 mmol/L increase)	1.28 (1.15, 1.42)	<0.001	1.30 (1.16, 1.45)	<0.001	1.25 (1.09, 1.44)	0.001	1.45 (1.19, 1.77)	<0.001
RPG (per 1 mmol/L increase)	1.10 (1.03, 1.17)	0.002	1.11 (1.04, 1.18)	0.002	1.13 (1.04, 1.24)	0.004	1.24 (1.10, 1.41)	0.001
HbA1c (per 1% increase)	0.97 (0.81, 1.16)	0.710	0.98 (0.81, 1.19)	0.845	1.23 (0.97, 1.55)	0.095	1.38 (1.00, 1.92)	0.053
**Glucose metrics threshold at admission**								
SHR1 ≥1.18	6.67 (3.70, 12.04)	<0.001	6.71 (3.67, 12.28)	<0.001	3.74 (1.77, 7.89)	0.001	5.38 (2.27, 12.76)	<0.001
SHR2 ≥1.26	3.21 (1.83, 5.61)	<0.001	3.28 (1.85, 5.81)	<0.001	2.47 (1.18, 5.16)	0.016	2.90 (1.23, 6.83)	0.015
SHR3 ≥1.01	6.17 (3.43, 11.11)	<0.001	6.04 (3.31, 11.00)	<0.001	2.99 (1.43, 6.28)	0.004	3.20 (1.43, 7.16)	0.005
SHR4 ≥1.03	3.04 (1.70, 5.42)	<0.001	2.94 (1.63, 5.30)	<0.001	2.09 (1.00, 4.38)	0.050	2.45 (1.08, 5.53)	0.031
GG ≥0.17	3.48 (1.92, 6.3)	<0.001	3.33 (1.82, 6.08)	<0.001	2.46 (1.16, 5.19)	0.019	2.72 (1.20, 6.19)	0.017
FPG ≥6.91 mmol/L	4.63 (2.60, 8.24)	<0.001	4.76 (2.63, 8.61)	<0.001	3.91 (1.85, 8.29)	<0.001	6.32 (2.51, 15.9)	<0.001
RPG ≥6.47 mmol/L	3.06 (1.66, 5.65)	<0.001	2.97 (1.60, 5.53)	0.001	2.74 (1.25, 5.98)	0.012	3.39 (1.39, 8.30)	0.007
HbA1c ≥5.55%	1.35 (0.75, 2.43)	0.318	1.24 (0.68, 2.27)	0.48	1.75 (0.78, 3.94)	0.173	1.42 (0.58, 3.48)	0.437
**Malignant cerebral edema** ^‡^ **(n = 52)**								
**Continuous glucose metrics at admission**								
SHR1 (per 1 point increase)	13.45 (5.48, 33.01)	<0.001	13.51 (5.50, 33.15)	<0.001	5.20 (1.93, 14.04)	0.001	4.73 (1.71, 13.04)	0.003
SHR2 (per 1 point increase)	3.04 (1.60, 5.78)	0.001	3.03 (1.59, 5.75)	0.001	1.84 (0.85, 4.00)	0.122	1.46 (0.64, 3.34)	0.373
SHR3 (per 1 point increase)	9.37 (3.66, 23.94)	<0.001	9.42 (3.68, 24.11)	<0.001	3.08 (1.25, 7.59)	0.014	3.21 (1.30, 7.94)	0.012
SHR4 (per 1 point increase)	2.48 (1.22, 5.02)	0.012	2.46 (1.22, 4.95)	0.012	1.52 (0.73, 3.15)	0.262	1.39 (0.64, 3.01)	0.408
GG (per 1 mmol/L increase)	1.18 (1.07, 1.31)	0.001	1.18 (1.07, 1.31)	0.001	1.09 (0.97, 1.23)	0.161	1.05 (0.93, 1.19)	0.446
FPG (per 1 mmol/L increase)	1.30 (1.16, 1.44)	<0.001	1.3 (1.17, 1.45)	<0.001	1.23 (1.08, 1.41)	0.002	1.20 (1.03, 1.40)	0.020
RPG (per 1 mmol/L increase)	1.08 (1.01, 1.15)	0.018	1.08 (1.01, 1.15)	0.018	1.08 (0.99, 1.17)	0.075	1.03 (0.94, 1.14)	0.534
HbA1c (per 1% increase)	1.01 (0.83, 1.21)	0.952	1.01 (0.83, 1.22)	0.930	1.17 (0.94, 1.46)	0.167	1.00 (0.75, 1.34)	0.976
**Glucose metrics threshold at admission**								
SHR1 ≥1.18	6.09 (3.22, 11.52)	<0.001	6.06 (3.20, 11.48)	<0.001	3.11 (1.48, 6.55)	0.003	2.72 (1.27, 5.84)	0.010
SHR2 ≥1.26	3.39 (1.84, 6.23)	<0.001	3.38 (1.83, 6.23)	<0.001	2.48 (1.19, 5.17)	0.016	2.09 (0.97, 4.51)	0.061
SHR3 ≥1.10	7.35 (3.9, 13.85)	<0.001	7.35 (3.90, 13.86)	<0.001	3.83 (1.83, 8.00)	<0.001	3.63 (1.72, 7.70)	0.001
SHR4 ≥1.15	3.30 (1.80, 6.04)	<0.001	3.32 (1.81, 6.08)	<0.001	2.35 (1.13, 4.89)	0.023	2.09 (0.99, 4.43)	0.053
GG ≥0.84	3.23 (1.76, 5.91)	<0.001	3.23 (1.76, 5.91)	<0.001	2.12 (1.02, 4.43)	0.044	1.81 (0.85, 3.87)	0.125
FPG ≥6.52 mmol/L	5.79 (2.91, 11.52)	<0.001	5.80 (2.91, 11.56)	<0.001	4.18 (1.9, 9.19)	<0.001	3.67 (1.58, 8.54)	0.002
RPG ≥6.47 mmol/L	3.75 (1.86, 7.59)	<0.001	3.73 (1.84, 7.55)	<0.001	3.27 (1.45, 7.38)	0.004	2.77 (1.17, 6.54)	0.020
HbA1c ≥4.65%	2.95 (0.38, 22.71)	0.300	2.90 (0.37, 22.43)	0.308	2.25 (0.18, 27.84)	0.526	1.78 (0.13, 23.80)	0.661
**Symptomatic intracerebral hemorrhage**^§^ **(n = 42)**								
**Continuous glucose metrics at admission**								
SHR1 (per 1 point increase)	12.59 (5.04, 31.45)	<0.001	12.80 (5.09, 32.16)	<0.001	5.20 (1.90, 14.27)	0.001	4.68 (1.48, 14.82)	0.009
SHR2 (per 1 point increase)	3.49 (1.77, 6.86)	<0.001	3.45 (1.76, 6.76)	<0.001	2.31 (1.06, 5.03)	0.034	2.34 (0.91, 6.05)	0.079
SHR3 (per 1 point increase)	8.90 (3.36, 23.57)	<0.001	9.12 (3.40, 24.50)	<0.001	3.24 (1.25, 8.36)	0.015	3.46 (1.32, 9.07)	0.012
SHR4 (per 1 point increase)	2.81 (1.32, 6.00)	0.007	2.78 (1.33, 5.83)	0.007	1.81 (0.88, 3.71)	0.107	2.07 (0.93, 4.62)	0.077
GG (per 1 mmol/L increase)	1.21 (1.09, 1.35)	<0.001	1.21 (1.09, 1.34)	0.001	1.14 (1.01, 1.28)	0.036	1.13 (0.98, 1.30)	0.104
FPG (per 1 mmol/L increase)	1.26 (1.13, 1.41)	<0.001	1.26 (1.13, 1.41)	<0.001	1.18 (1.04, 1.35)	0.011	1.12 (0.95, 1.33)	0.164
RPG (per 1 mmol/L increase)	1.08 (1.01, 1.16)	0.020	1.08 (1.01, 1.16)	0.021	1.08 (1.00, 1.18)	0.057	1.06 (0.95, 1.19)	0.321
HbA1c (per 1% increase)	0.96 (0.77, 1.19)	0.707	0.96 (0.76, 1.20)	0.711	1.08 (0.84, 1.39)	0.539	0.91 (0.63, 1.32)	0.630
**Glucose metrics threshold at admission**								
SHR1 ≥1.12	8.15 (3.75, 17.72)	<0.001	7.90 (3.63, 17.21)	<0.001	4.93 (2.12, 11.46)	<0.001	4.30 (1.68, 11.05)	0.002
SHR2 ≥1.20	4.30 (2.08, 8.88)	<0.001	4.20 (2.03, 8.7)	<0.001	3.85 (1.70, 8.70)	0.001	3.06 (1.25, 7.50)	0.015
SHR3 ≥1.10	7.49 (3.76, 14.92)	<0.001	7.59 (3.79, 15.2)	<0.001	4.21 (1.93, 9.20)	<0.001	4.24 (1.75, 10.26)	0.001
SHR4 ≥1.03	3.24 (1.60, 6.57)	0.001	3.16 (1.55, 6.42)	0.001	2.34 (1.06, 5.16)	0.036	1.86 (0.78, 4.44)	0.161
GG ≥0.45	3.46 (1.75, 6.85)	<0.001	3.39 (1.71, 6.72)	<0.001	2.41 (1.11, 5.21)	0.025	2.21 (0.93, 5.22)	0.071
FPG ≥6.63 mmol/L	7.46 (3.33, 16.68)	<0.001	7.30 (3.26, 16.37)	<0.001	5.61 (2.33, 13.48)	<0.001	4.62 (1.73, 12.36)	0.002
RPG ≥6.47 mmol/L	4.17 (1.87, 9.30)	<0.001	4.05 (1.81, 9.04)	0.001	3.52 (1.46, 8.53)	0.005	3.10 (1.13, 8.46)	0.027
HbA1c ≥6.45%	1.24 (0.60, 2.54)	0.561	1.21 (0.59, 2.51)	0.600	2.28 (0.96, 5.44)	0.062	1.47 (0.46, 4.75)	0.519
**3-month mortality**^†^ **(n = 83)**								
**Continuous glucose metrics at admission**								
SHR1 (per 1 point increase)	16.39 (6.71, 40.05)	<0.001	18.39 (7.38, 45.85)	<0.001	9.61 (3.31, 27.91)	<0.001	10.87 (3.56, 33.21)	<0.001
SHR2 (per 1 point increase)	4.38 (2.32, 8.25)	<0.001	4.73 (2.48, 9.05)	<0.001	4.12 (1.70, 10.01)	0.002	4.74 (1.87, 12.05)	0.001
SHR3 (per 1 point increase)	11.36 (4.60, 28.03)	<0.001	12.43 (4.92, 31.42)	<0.001	5.04 (2.09, 12.19)	<0.001	4.54 (1.73, 11.87)	0.002
SHR4 (per 1 point increase)	3.97 (1.92, 8.20)	<0.001	4.10 (1.95, 8.64)	<0.001	2.97 (1.45, 6.07)	0.003	2.61 (1.25, 5.48)	0.011
GG (per 1 mmol/L increase)	1.27 (1.15, 1.41)	<0.001	1.30 (1.17, 1.44)	<0.001	1.25 (1.08, 1.44)	0.003	1.32 (1.11, 1.57)	0.002
FPG (per 1 mmol/L increase)	1.31 (1.18, 1.45)	<0.001	1.34 (1.20, 1.49)	<0.001	1.34 (1.16, 1.55)	<0.001	1.68 (1.35, 2.08)	<0.001
RPG (per 1 mmol/L increase)	1.10 (1.03, 1.16)	0.002	1.11 (1.04, 1.18)	0.001	1.14 (1.04, 1.24)	0.003	1.25 (1.11, 1.42)	<0.001
HbA1c (per 1% increase)	0.96 (0.81, 1.13)	0.588	0.95 (0.80, 1.14)	0.601	1.18 (0.94, 1.48)	0.154	1.32 (0.97, 1.79)	0.074
**Glucose metrics threshold at admission**								
SHR1 ≥1.18	7.48 (4.35, 12.87)	<0.001	7.60 (4.32, 13.37)	<0.001	5.78 (2.78, 12.04)	<0.001	8.11 (3.44, 19.09)	<0.001
SHR2 ≥1.26	3.46 (2.08, 5.76)	<0.001	3.65 (2.14, 6.22)	<0.001	3.24 (1.58, 6.65)	0.001	4.72 (2.00–11.14)	<0.001
SHR3 ≥1.03	6.65 (3.88, 11.38)	<0.001	6.98 (3.98, 12.25)	<0.001	4.15 (2.03, 8.46)	<0.001	4.41 (2.04, 9.55)	<0.001
SHR4 ≥1.03	3.79 (2.22, 6.48)	<0.001	3.84 (2.21, 6.67)	<0.001	3.61 (1.74, 7.47)	0.001	4.49 (2.01, 10.04)	<0.001
GG ≥0.17	4.21 (2.44, 7.28)	<0.001	4.20 (2.40, 7.36)	<0.001	4.03 (1.93, 8.42)	<0.001	4.79 (2.13, 10.74)	<0.001
FPG ≥6.52 mmol/L	4.86 (2.83, 8.34)	<0.001	4.84 (2.77, 8.43)	<0.001	5.58 (2.63, 11.83)	<0.001	9.75 (3.87, 24.54)	<0.001
RPG ≥6.46 mmol/L	2.99 (1.74, 5.16)	<0.001	2.91 (1.66, 5.08)	<0.001	2.85 (1.37, 5.92)	0.005	3.49 (1.51–8.03)	0.003
HbA1c ≥4.95%	1.66 (0.67, 4.14)	0.277	1.30 (0.51, 3.37)	0.583	1.13 (0.30, 4.24)	0.857	0.91 (0.22, 3.70)	0.893
**3-month poor functional outcome**^†^ **(n = 138)**								
**Continuous glucose metrics at admission**								
SHR1 (per 1 point increase)	16.17 (6.48, 40.31)	<0.001	16.57 (6.48, 42.36)	<0.001	7.61 (2.81, 20.63)	<0.001	8.05 (2.77, 23.39)	<0.001
SHR2 (per 1 point increase)	3.35 (1.82, 6.18)	<0.001	3.34 (1.80, 6.20)	<0.001	2.78 (1.25, 6.17)	0.012	2.73 (1.20, 6.22)	0.017
SHR3 (per 1 point increase)	10.11 (4.11, 24.87)	<0.001	10.31 (4.08, 26.06)	<0.001	3.82 (1.62, 8.98)	0.002	3.76 (1.41, 10.04)	0.008
SHR4 (per 1 point increase)	2.82 (1.43, 5.58)	0.003	2.74 (1.39, 5.41)	0.004	2.05 (1.04, 4.06)	0.039	1.79 (0.89, 3.59)	0.100
GG (per 1 mmol/L increase)	1.22 (1.11, 1.34)	<0.001	1.22 (1.11, 1.35)	<0.001	1.17 (1.03, 1.33)	0.014	1.18 (1.03, 1.35)	0.014
FPG (per 1 mmol/L increase)	1.34 (1.20, 1.49)	<0.001	1.35 (1.21, 1.51)	<0.001	1.30 (1.14, 1.48)	<0.001	1.51 (1.26, 1.80)	<0.001
RPG (per 1 mmol/L increase)	1.09 (1.03, 1.16)	0.003	1.10 (1.03, 1.17)	0.004	1.10 (1.02, 1.18)	0.012	1.14 (1.04, 1.26)	0.005
HbA1c (per 1% increase)	1.01 (0.88, 1.16)	0.858	1.01 (0.87, 1.17)	0.885	1.13 (0.95, 1.34)	0.156	1.19 (0.95, 1.50)	0.130
**Glucose metrics threshold at admission**								
SHR1 ≥1.18	5.88 (3.56, 9.72)	<0.001	6.14 (3.60, 10.49)	<0.001	4.25 (2.21, 8.20)	<0.001	5.11 (2.52, 10.37)	<0.001
SHR2 ≥1.15	3.01 (1.90, 4.76)	<0.001	2.72 (1.68, 4.39)	<0.001	2.43 (1.32, 4.45)	0.004	2.67 (1.40, 5.11)	0.003
SHR3 ≥1.01	4.81 (2.95, 7.83)	<0.001	4.86 (2.90, 8.15)	<0.001	2.89 (1.52, 5.49)	<0.001	3.17 (1.62, 6.20)	0.001
SHR4 ≥1.03	2.33 (1.49, 3.64)	<0.001	2.27 (1.42, 3.63)	<0.001	1.93 (1.05, 3.54)	0.033	1.99 (1.06, 3.72)	0.031
GG ≥0.17	2.43 (1.55, 3.79)	<0.001	2.35 (1.47, 3.76)	<0.001	2.00 (1.09, 3.67)	0.024	2.04 (1.09, 3.81)	0.025
FPG ≥6.50 mmol/L	3.94 (2.48, 6.24)	<0.001	3.84 (2.36, 6.23)	<0.001	3.68 (1.99, 6.80)	<0.001	5.69 (2.68, 12.07)	<0.001
RPG ≥6.46 mmol/L	2.48 (1.58, 3.91)	<0.001	2.37 (1.47, 3.81)	<0.001	2.02 (1.11, 3.67)	0.021	2.28 (1.16, 4.47)	0.017
HbA1c ≥5.65%	0.96 (0.56, 1.65)	0.889	0.90 (0.51, 1.59)	0.721	1.36 (0.67, 2.75)	0.394	1.34 (0.48, 3.73)	0.577

**Abbreviations:** AOR, adjusted odds ratio; FPG, fasting plasma glucose; Glycated hemoglobin, HbA1c; GG, glycemic gap; OR: odds ratio; RPG, random plasma glucose; SHR, stress hyperglycemia ratio; SHR1, [FPG (mmol/L)]/[HbA1c (%)]; SHR2, [admission RPG (mmol/L)]/[HbA1c (%)]; SHR3, FPG (mmol/L)/[(1.59 × HbA1c)−2.59]; SHR4, [admission RPG (mmol/L)]/[(1.59 × HbA1c)−2.59]; GG, admission RPG − [(1.59 × HbA1c)−2.59].

^†^Variables adjusted for are as follows: model A is age and sex; model B is model A+ TOAST classification, NIHSS, and baseline ASPECTS ≤7; model C is model B + comorbidities (DM, CKD, MI, CHF, preexisting disability, and history of malignancy)

^‡^Variables adjusted for are as follows: model A is age and sex; model B is model A+ TOAST classification, NIHSS, baseline ASPECTS ≤7, SBP, and DBP; model C is model B + comorbidities (Hypertension and DM)

^§^Variables adjusted for are as follows: model A is age and sex; model B is model A+NIHSS, baseline ASPECTS ≤7, SBP, and DBP; model C is model B + comorbidities (Hypertension, DM, prior use antiplatelet, onset to treatment time, and antihypertensive before rt-PA)

#### 3.3.2 MCE

According to the MVLR analysis, we found that SHR1, SHR3, and FPG were associated with the development of MCE after adjusting for age, sex, TOAST classification, NIHSS, baseline ASPECTS ≤7, SBP, DBP, hypertension, and DM. Each one-point increase in SHR1, SHR3, and FPG was associated with MCE with odds ratios of 4.73 (95% CI: 1.71, 13.04; *p* =  0.003), 3.21 (95% CI: 1.30, 7.94; *p* =  0.012), and 1.20 (95% CI: 1.03, 1.40; *p* =  0.020), respectively. However, HbA1c was not associated with the outcome in any model. SHR1 ≥1.18, SHR3 ≥1.10, FPG ≥ 6.52 mmol/L, and admission RPG ≥ 6.47 mmol/L were associated with MCE after adjusting for the aforementioned variables. In Model C, SHR2, SHR4, GG, and HbA1c were not associated with MCE in either a continuous or threshold model. Additionally, SHR1 had the strongest association with MCE compared to other continuous glucose metrics (Table 3).

#### 3.3.3 sICH

We found that both SHR1 and SHR3 in both continuous and threshold glucose metrics were associated with sICH after adjusting for age, sex, NIHSS, baseline ASPECTS ≤7, SBP, DBP, comorbidities (hypertension and DM), prior taking antiplatelet medication, onset-to-treatment time, and antihypertensive medication before rt-PA. In model C, an FPG level of ≥6.63 mmol/L demonstrated the highest association with sICH among the threshold glucose metrics (OR = 4.62, 95% CI [1.73–12.36]; *p* = 0.002). Among the various continuous glucose metrics, SHR1 exhibited the strongest link with sICH (per 1 point increase: OR = 4.68, 95% CI [1.48–14.82]; *p* = 0.009) (Table 3).

#### 3.3.4 Mortality at 3 months

We found that SHR1, SHR2, SHR3, SHR4, GG, FPG, and RPG in both continuous and threshold glucose metrics were significantly associated with 3-month mortality in all models. In the MVLR analysis, the SHR1 in continuous glucose metrics was most strongly associated with 3-month mortality in model C (per 1 mmol/L increase): OR 10.87, 95% CI 3.56–1.25, *p*<0.001). SHR1≥ 1.18 had an 8.11-fold higher risk of 3-month mortality (OR 8.11, 95% CI 3.44–19.09, *p*< 0.001) (Table 3).

#### 3.3.5 Poor functional outcome at 3 months

We performed univariate and MVLR analyses to examine the association between various glucose metrics and 3-month poor functional outcomes. SHR1, SHR2, SHR3, SHR4, GG, FPG, and RPG in both continuous and threshold glucose metrics were consistently associated with outcomes across all models. For continuous glucose metrics in model C, SHR1 showed the strongest association with 3-month poor functional outcomes (OR 8.05, 95% CI 2.77–23.39, *p*< 0.001). In the threshold analysis for model C, both SHR1 (≥1.18) and FPG (≥6.50 mmol/L) were significantly associated with 3-month poor functional outcomes (OR 5.11, 95% CI 2.52–10.37, *p*< 0.001, and OR 5.69, 95% CI 2.68–12.07, *p*< 0.001, respectively) (Table 3).

SHR1 in continuous glucose metrics remained a striking predictor and had the greatest impact on fatal outcomes after thrombolysis. The association between FPG in glucose metric threshold had the strongest relationship with fatal outcomes, which was further strengthened after adjusting for fully adjusted Model C. The distribution of mRS score at time of hospital discharge and 3-month follow-up in group stratified according to cut-off value of SHR1. (S1 Fig). IHM, MCE, 3-month mortality, and poor functional outcome rates were higher in patients with SHR1≥1.18 compared to those with SHR1<1.18 (39.4% versus 8.9%; *p*<0.001, 32.1% versus 7.2%; *p*<0.001, 50.5% versus 11.9%; *p*<0.001, and 69.4% versus 27.9%; *p*<0.001, respectively), while the sICH rate was higher in patients with SHR1≥1.12 compared to those with SHR1<1.12 (26.0% versus 4.1%; *p*<0.001) (S2 Fig).

### 3.4 Results of subgroup analysis for the fatal outcomes

Surprisingly, we also found that elevated SHR1 (SHR1≥1.18 [for IHM, MCE, 3-month mortality, and poor functional outcome] and ≥1.12 [for sICH]) was independently associated with fatal outcomes in non-DM AIS patients. The ORs for IHM were 6.13 versus 3.36 (*p* < 0.001), for MCE were 4.29 versus 0.84 (*p*<0.001), for 3-month mortality were 14.9 versus 11.6 (*p* = 0.014), and for 3-month poor functional outcome were 6.48 versus 5.36 (*p* = 0.003) compared with those who had DM. Similarly, elevated SHR1 with SBP≥180 mmHg and NIHSS<16 were associated with an increased 3-month mortality risk and poor functional outcome compared with those with SBP<180 mmHg and NIHSS≥16, respectively (see S1 Table).

## 4. Discussion

The relationship between the SHR and fatal outcomes in AIS patients treated with rt-PA was identified in our cohort study. The main findings of this study were as follows: (1) SHR1 had the greatest predictive power for fatal outcomes among other glucose metrics; (2) SHR1, as a continuous variable, had the strongest association with fatal outcomes after adjustment for potential confounders; (3) both SHR1 and SHR3 at admission, as continuous variables and at thresholds, were independently associated with impact on fatal outcomes in the MVLR model, indicating that these indicators might have an important role in glycemic control intervention in patients with SH; (4) in subgroup analysis, elevated SHR1 had a stronger association with IHM, MCE, 3-month mortality, and poor functional outcome in patients aged ≥ 70 years compared to those < 70 years., while elevated SHR1 had a stronger association with sICH in patients < 70 years compared to those ≥ 70 years. Furthermore, elevated SHR1 was more strongly associated with fatal outcomes in AIS patients who were non-DM, with SBP≥ 180 mmHg, and NIHSS<16.

To our knowledge, there were few studies discussing the association between SHR and fatal outcomes in thrombolyzed AIS patients. Our study findings aligned with previous research, indicating that SHR1 was a significant predictor of 3-month mortality and sICH in AIS patients who underwent both rt-PA treatment [21–24] and EVT [25]. The mechanism of the relationship between SH and fatal outcomes is not yet clearly understood. Several mechanisms have been proposed. Firstly, SH may serve as a marker indicating the extent of ischemic damage following stroke. SH is characterized by rapid blood glucose elevation due to hypothalamic-pituitary-adrenal axis and sympathetic nervous system activation, which promotes excessive gluconeogenesis, glycogenolysis, and insulin resistance through the complex interaction of hormones, including cortisol, growth hormone, glucagon, and catecholamines. [26, 27]. The hyperglycemic state leads to a dramatic elevation of inflammatory cytokines and vasoconstrictive factors, contributing to fatal outcomes [28]. Secondly, AIS patients’ acute stress-related inflammation leads to rapid accumulation of circulating free fatty acids and oxidative stress. These contribute to the decline in endothelial nitric oxide, a vasodilator, and an increase in plasminogen activator inhibitor, further worsening penumbra perfusion and ischemia [29]. Thirdly, SH is related to the reperfusion injury after successful recanalization, which increases the risk of hemorrhagic transformation, one of the fatal outcomes in AIS patients [30].

We speculate that FPG represent the genuine blood glucose level affected from the stress without being confounded by meal-derived glucose as the RPG [31]. Consequently, SHR1 and SHR3 which were calculated from FPG represented stronger association and higher predictive values to the fatal outcomes than SHR2 and SHR4. Increasing hyperglycemia in AIS patients escalates oxidative stress, neurohormonal derangement, and inflammatory cytokines, thereby perpetuating a vicious cycle that exacerbates hyperglycemia [23].

Meta-analyses have shown that acute hyperglycemia is related to IHM and poor functional recovery in non-DM AIS patients [28]. In a recent study, Merlino et al. demonstrated that diabetic status has a protective role on the decremental effects of SH in AIS patients receiving alteplase. Conversely, non-DM with severe SH had a higher incidence of poor outcomes at three months and sICH. [32] This is consistent with our study’s subgroup analysis, demonstrating that elevated SHR1 was more strongly associated with fatal outcomes in non-DM patients than in DM AIS patients. The relationship between SH and poor outcomes in non-DM AIS patients has several explanations. First, the glycemic threshold for stress hyperglycemia on top chronic hyperglycemia in DM is higher than that in normoglycemic patients. Second, chronic hyperglycemia in DM patients affects their physiologic response, so they may develop tolerance mechanisms that diminish the deleterious metabolic effects [21]. Third, acute hyperglycemia in non-DM individuals may indicate a more severe or prolonged stress response, which can contribute to poorer clinical outcomes. Lastly, DM patients tend to receive more intensive glycemic management due to healthcare providers’ awareness.

Optimal glycemic control benefits survival in both DM and non-DM AIS patients by mitigating cerebral lactic acidosis and other detrimental metabolic processes that expedite ischemic brain damage. Nonetheless, more research is required to ascertain the clinical advantages of aggressive insulin treatment versus standard care. Animal models have suggested that lowering blood sugar with insulin can reduce ischemic brain damage [33]. Previous studies, including the UK Glucose Insulin in Stroke Trial [34], have demonstrated that insulin infusions lead to a significant decrease in plasma glucose levels compared to the saline group. However, despite this reduction, no noticeable improvement in clinical outcomes was observed. The SHINE study [35] categorized AIS patients into two groups: those treated with subcutaneous insulin on a sliding scale and those treated with continuous intravenous insulin. The study found no significant difference in 90-day functional outcomes between the two treatment groups, but it is possible that the study was underpowered.

There are some cautions in determining SHR in AIS patients. Based on several previous studies [36–38], SH is defined as absolute hyperglycemia without deterioration of pre-illness glycemic control in patients with pre-existing DM, without considering background glucose levels, which makes it difficult to distinguish SH in DM patients. Relative hyperglycemia might be a better predictor of critical disease outcomes than absolute hyperglycemia [39]. However, CKD, anemia, and hemoglobinopathies could affect HbA1c measurement accuracy [40]. Therefore, it is important to consider the interpretation of SHR in patients with these conditions.

The research had some limitations. Firstly, it was a single-center, retrospective observational study, which emphasized the importance of conducting multi-site clinical trials. Secondly, we did not determine the glycemic variability in this study due to lack of precise information and retrospective nature. Thirdly, the study had a small number of DM patients, which may have resulted in low power to detect the same relative risk in DM patients as in non-DM patients. Lastly, the study included only Thai patients; therefore, these findings cannot be generalized to other ethnicities.

## 5. Conclusions

In summary, our study demonstrated that both SHR1 and SHR3 are independently associated with fatal outcomes. Notably, SHR1 emerged as the most valuable biomarker for predicting fatal outcomes following rt-PA treatment. We recommend close monitoring of patients with elevated SHR1 levels upon admission, especially in non-DM, SBP≥ 180 mmHg, and NIHSS<16. Future studies are required to determine whether enhancing clinical prediction models with the combination of SHR and traditional risk factors enhances the accuracy of predicting fatal ischemic stroke outcomes.

## Supporting information

S1 Fig Functional outcome at (A) the time of hospital discharge and (B) the 3-month follow-up in groups stratified according to the cut-off value of SHR1.
(TIF)

S2 Fig Fatal outcomes in groups stratified according to the cut-off value of SHR1. Note: This figure shows a comparison between SHR1≥ 1.18 for IHM, MCE, 3-month mortality, and poor functional outcome; and SHR1≥ 1.12 for sICH. The numbers next to the bar graphs reflect the percentage of each fatal outcome within this group. *P*-values were calculated using Pearson chi-square/Fisher exact tests where appropriate.
(TIF)

S1 Table Subgroup analyses for the risk of in-hospital mortality by SHR1≥1.18 (for IHM, MCE, 3-month mortality and poor functional outcome) and ≥1.12 (for sICH). (DOCX)
