# Supplementary figures and images for: Performance comparison of stress hyperglycemia ratio for predicting fatal outcomes in patients with thrombolyzed acute ischemic stroke
Source: PLoS One. 2024 Jan 31;19(1):e0297809. doi: 10.1371/journal.pone.0297809 (PMC10830025; doi:10.1371/journal.pone.0297809)

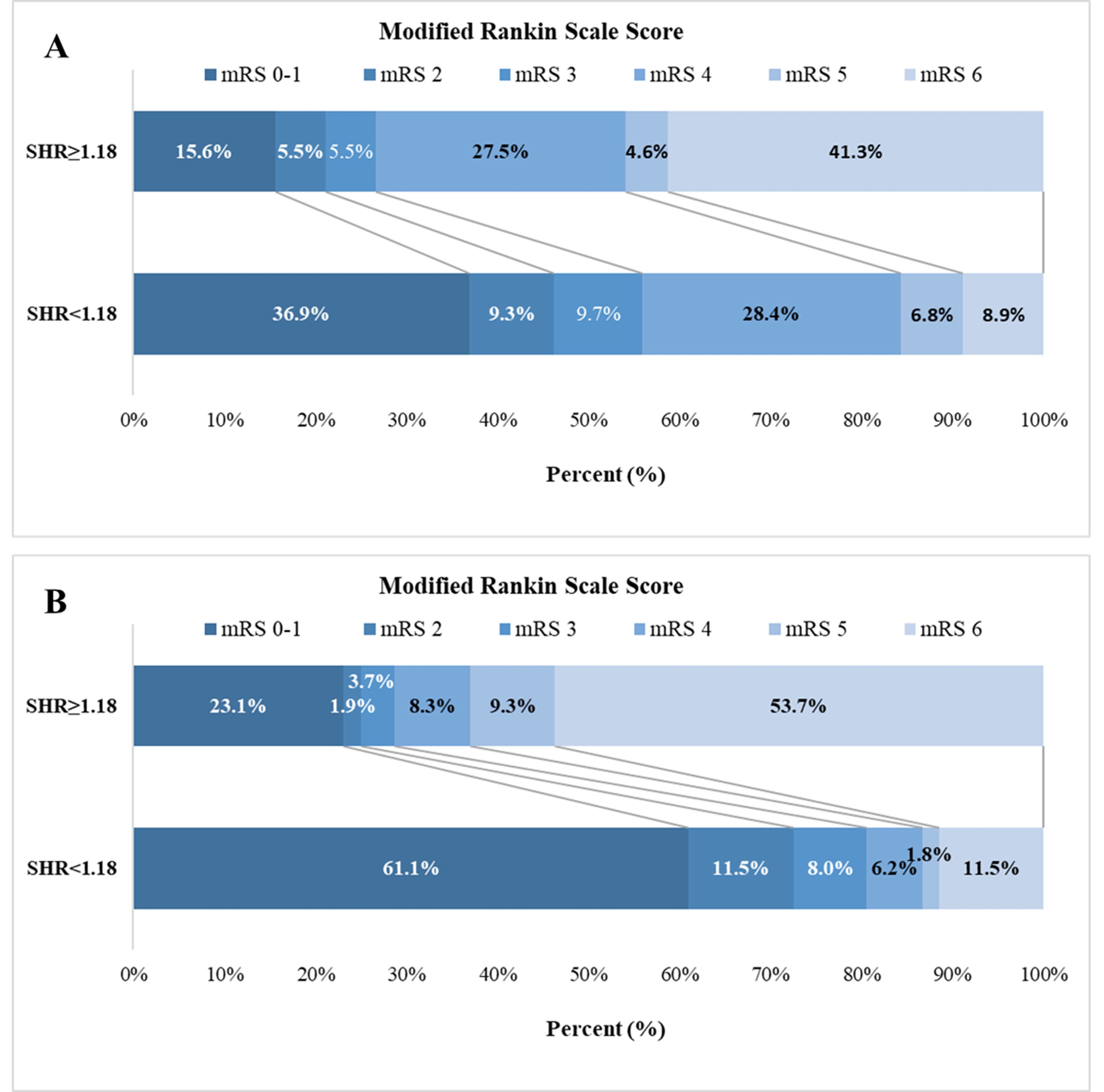

Supplement: S1 Fig — Functional outcome at (A) the time of hospital discharge and (B) the 3-month follow-up in groups stratified according to the cut-off value of SHR1. (TIF) [file pone.0297809.s001.tif]

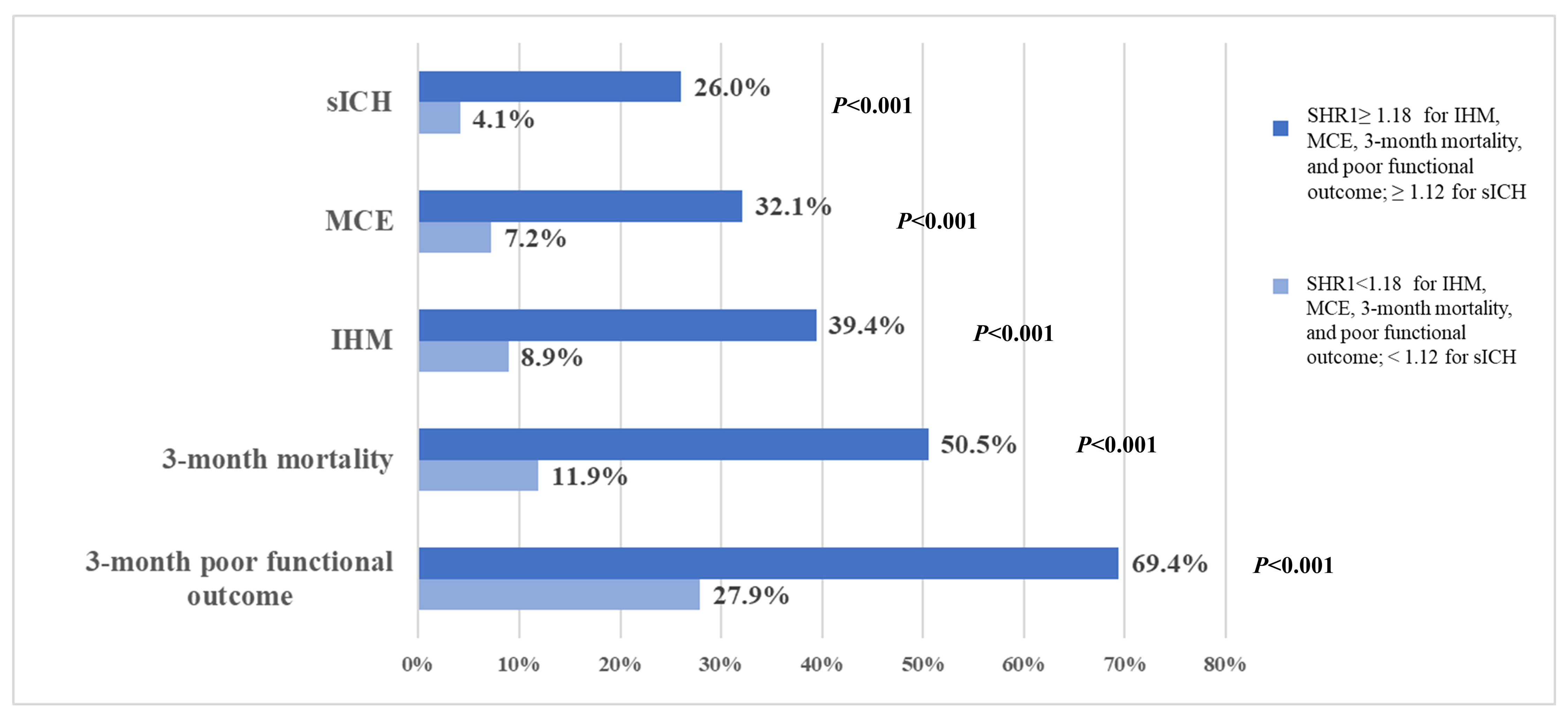

Supplement: S2 Fig — Note: This figure shows a comparison between SHR1≥ 1.18 for IHM, MCE, 3-month mortality, and poor functional outcome; and SHR1≥ 1.12 for sICH. The numbers next to the bar graphs reflect the percentage of each fatal outcome within this group. P-values were calculated using Pearson chi-square/Fisher exact tests where appropriate. (TIF) [file pone.0297809.s002.tif]
