# Supplementary material for: Performance comparison of stress hyperglycemia ratio for predicting fatal outcomes in patients with thrombolyzed acute ischemic stroke
Source: PLoS One. 2024 Jan 31;19(1):e0297809. doi: 10.1371/journal.pone.0297809 (PMC10830025; doi:10.1371/journal.pone.0297809)
Supplement: S1 Table — (DOCX) [file pone.0297809.s003.docx]

**Supporting information**

**S1 Table.** Subgroup analyses for the risk of in-hospital mortality by SHR1≥1.18 (for IHM, MCE, 3-month mortality and poor functional outcome) and ≥1.12 (for sICH).

| Variables | Primary outcome | Secondary outcome | | | |
| --- | --- | --- | --- | --- | --- |
|  | IHM ^†^ (n=65)  AOR (95% CI) | MCE^‡^ (n=52)  AOR (95% CI) | sICH^§^ (n=42)  AOR (95% CI) | 3-month mortality risk^†^  (n=83)  AOR (95% CI) | 3-month poor functional outcome^†^ (n=138)  AOR (95% CI) |
| Age | | | |  |  |
| ≥ 70 years | 8.29 (1.87, 36.79) | 3.17 (0.74, 13.47) | 2.05 (0.38, 11.25) | 9.06 (3.36, 24.42) | 6.18 (2.82, 13.56) |
| < 70 years | 4.15 (1.23, 14.01) | 2.25 (0.84, 6.01) | 5.88 (1.68, 20.57) | 7.69 (2.55, 23.16) | 4.21 (1.43, 12.33) |
| *p*-value for interaction† | <0.001 | <0.001 | <0.001 | <0.001 | <0.001 |
| Sex | | | |  |  |
| Male | 6.13 (1.71, 21.96) | 1.54 (0.45, 5.23) | 5.29 (0.98, 28.61) | 11.7 (4.04, 33.9) | 6.16 (2.55, 14.86) |
| Female | 10.76 (2.36, 49.15) | 4.13 (1.38, 12.36) | 6.16 (1.47, 25.94) | 7.12 (2.49, 20.37) | 5.06 (2.05, 12.49) |
| *p*-value for interaction† | <0.001 | <0.001 | <0.001 | <0.001 | <0.001 |
| DM | | | |  |  |
| Yes | 3.36 (0.34, 33.49) | 0.84 (0.19, 3.62) | 1.39 (0.22, 8.59) | 11.6 (4.65, 28.95) | 5.36 (2.54, 11.33) |
| No | 6.13 (2.26, 16.6) | 4.29 (1.66, 11.09) | 5.96 (1.9, 18.67) | 14.9 (1.95, 113.84) | 6.48 (1.95, 21.59) |
| *p*-value for interaction† | <0.001 | <0.001 | <0.001 | 0.014 | 0.003 |
| SBP | | | |  |  |
| ≥ 180 mmHg | 6.10 (0.87, 42.98) | 3.86 (0.75, 19.99) | 10.85 (1.52, 77.47) | 19.8 (3.57, 109.98) | 10.3 (2.42, 43.85) |
| < 180 mmHg | 4.27 (1.44, 12.67) | 2.62 (1.02, 6.73) | 3.56 (1.11, 11.41) | 5.32 (2.26, 12.55) | 3.96 (1.94, 8.05) |
| *p*-value for interaction† | <0.001 | <0.001 | <0.001 | <0.001 | <0.001 |
| NIHSS at admission | | | |  |  |
| ≥16 | 4.75 (1.51, 14.88) | 1.57 (0.57, 4.31) | 2.49 (0.7, 8.86) | 5.34 (2.05, 13.93) | 3.95 (1.21, 12.89) |
| <16 | 21.69 (2.29, 205.37) | 7.68 (1.68, 35.21) | 28.07 (2.19, 359.43) | 22.56 (4.3, 118.43) | 4.82 (2.25, 10.36) |
| *p*-value for interaction† | <0.001 | <0.001 | <0.001 | <0.001 | <0.001 |

**Abbreviations:** AOR, adjusted odds ratio; CHF, congestive heart failure; CKD, chronic kidney disease; DBP, diastolic blood pressure; DM, diabetes mellitus; HT, hypertension; MI, myocardial infarction; NIHSS, National Institutes of Health Stroke Scale; rt-PA; recombinant tissue plasminogen activator; SHR, stress hyperglycemia ratio; SHR1, [FPG (mmol/L)]/[HbA1c (%)];SBP, systolic blood pressure; TOAST, Trial of Org 10172 in Acute Stroke Treatment.

^†^Variables adjusted for are as follows: age, sex, TOAST classification, NIHSS, baseline ASPECTS ≤7, DM, CKD, MI, CHF, preexisting disability, and history of malignancy

^‡^Variables adjusted for are as follows: age, sex, TOAST classification, NIHSS, baseline ASPECTS ≤7, SBP, DBP, HT, DM

^§^Variables adjusted for are as follows: age, sex, NIHSS, baseline ASPECTS ≤7, SBP, DBP, HT, DM, prior use antiplatelet, onset to treatment time, antihypertensive before rt-PA.

In each case, the model is not adjusted for the stratification variable.

†We tested for an interaction (ie, if the effect of elevated SHR1 was the same for both subgroups) using a logistic regression

model with an interaction term between the strata (for example, age <70 vs age ≥ 70 years) and elevated SHR1.
